# Galangin and Pinocembrin from Propolis Ameliorate Insulin Resistance in HepG2 Cells via Regulating Akt/mTOR Signaling

**DOI:** 10.1155/2018/7971842

**Published:** 2018-10-21

**Authors:** Yinkang Liu, Xiali Liang, Gensheng Zhang, Lingjie Kong, Wenjun Peng, Hongcheng Zhang

**Affiliations:** ^1^Institute of Apicultural Research, Chinese Academy of Agricultural Sciences, Beijing 100093, China; ^2^College of Food Engineering, Harbin University of Commerce, Harbin 150076, China; ^3^National Research Center of Bee Product Processing, Ministry of Agriculture, Beijing 100093, China

## Abstract

Insulin resistance has a critical role in type 2 diabetes. The aim of this study was to investigate the effect of pinobanksin, galangin, chrysin, and pinocembrin from propolis on insulin resistance. Our study shows that galangin and pinocembrin can ameliorate insulin resistance; on the contrary, pinobanksin and chrysin are ineffective. Galangin and pinocembrin treatments substantially increase glucose consumption and glycogen content by enhancing the activities of hexokinase and pyruvate kinase. Galangin treatment with 80 *μ*M increased hexokinase and pyruvate kinase activities by 21.94% and 29.12%, respectively. Moreover, we hypothesize that galangin and pinocembrin may have a synergistic effect on the improvement of insulin resistance via Akt/mTOR signaling pathway, through distinctly upregulating the phosphorylation of IR, Akt, and GSK3*β* and remarkably downregulating the phosphorylation of IRS. Most notably, this is the first study to our knowledge to investigate pinocembrin about the alleviation of insulin resistance. Our results provide compelling evidence for the depth development of propolis products to ameliorate insulin resistance.

## 1. Introduction

Diabetes mellitus refers to a group of serious chronic metabolic disorders, characterized by hyperglycemia. Nowadays, diabetes mellitus has emerged as a major global public health problem. The prevalence and incidence of diabetes mellitus have been experiencing rapid growth over recent decades. It is estimated that the total number of diabetes aged 20–79 years will rise from 415 million in 2015 to 642 million in 2040 [1]. Moreover, approximately 90–95% of diabetic individuals suffer from type 2 diabetes mellitus (T2DM). T2DM is characterized by impairment in pancreatic *β*-cell and insulin resistance in target organs. Insulin resistance can cause many severe complications, for example, hypertension, coronary heart disease, and so on. Thus, the treatments of insulin resistance seem to be worthy of more attention and investigation.

Propolis is often defined as natural complex substance collected by* Apis mellifera* and widely used as the medical and healthcare due to a wide range of biological properties. Indeed, several studies have demonstrated that propolis possesses antimicrobial [2], antioxidative [3], anti-inflammatory [4], and antitumor [5] capacities. In recent years, a strong relationship between propolis and insulin resistance has been reported in the literatures. For instance, Chinese propolis can have an appreciable impact on controlling blood glucose, modulating lipid metabolism, and improving insulin sensitivity in T2DM rats [6]. In addition, the extract of Brazilian propolis did not impact body weight gain and food intake in ob/ob mice, but insulin resistance was significantly reduced [7]. Overall, the findings indicate that propolis seem to play an essential role in the treatment of insulin resistance. However, these studies have tended to focus on the effect of propolis or propolis extract rather than individual propolis compounds.

Flavonoids present widely in certain food and healthcare products and have been applied in ameliorating insulin resistance. Previous research has noted that flavonoids play a critical role in signaling pathways related to insulin resistance. For example, cocoa flavonoids can modulate AMPK signaling pathway to relieve insulin resistance of high glucose-treated HepG2 cells [8]. In addition, quercetin can also regulate AMPK signaling to alleviate high fructose-induced insulin resistance in humans [9]. Besides AMPK signaling pathway, other signaling pathways seem to be related to insulin resistance, including endoplasmic reticulum (ER) stress [10], mitogen-activated protein kinase (MAPK) [11], and protein kinases B/mammalian target of rapamycin (Akt/mTOR) [12, 13]. Akt/mTOR is an important pathway of intracellular insulin transduction and energy metabolism in the liver and plays a very central role in glycolysis [14, 15], gluconeogenesis [14], and glycogen synthesis [16]. However, it remains unclear whether propolis flavonoids ameliorate insulin resistance via Akt/mTOR signaling pathway.

In this paper, based on their specific and abundance in propolis, we selected pinobanksin, galangin, chrysin, and pinocembrin to investigate their effects on insulin resistance and Akt/mTOR signaling. In addition, variations of glucose consumption, glycogen content, hexokinase, and pyruvate kinase activities were measured in insulin-resistant HepG2 cells by enzyme immunoassay kit. The phosphorylation and dephosphorylation of key Akt/mTOR signaling proteins were tested using the MILLIPLEX® _MAP_ Akt/mTOR 11-plex Panel.

## 2. Materials and Methods

### 2.1. Materials

Dulbecco's modified eagle medium (DMEM), phosphate buffered saline (PBS), hexokinase (HK) assay kit, and pyruvate kinase (PK) assay kit were from Solarbio (Beijing, China). Dimethyl sulfoxide (DMSO), 3-(4,5-dimethyl-2-thiazolyl)-2,5-diphenyl-2-H-tetrazolium bromide (MTT), trypsin-EDTA solution 1× (0.25% trypsin, 0.02% EDTA), insulin solution (human), bicinchoninic acid (BAC) protein assay kit, pinobanksin, galangin, chrysin, and pinocembrin were obtained from Sigma-Aldrich (St. Louis, MO, USA). Fetal bovine serum (FBS) was obtained from Gibco (Grand Island, NY, USA). Glucose assay kit and glycogen assay kit were purchased from BioVision (Milpitas, CA, USA). MILLIPLEX® _MAP_ Akt/mTOR Phosphoprotein 11-plex Magnetic Bead Kit and MILLIPLEX® _MAP_ Akt/mTOR Total Protein 11-plex Magnetic Bead Kit were purchased from Merck Millipore (Darmstadt, Germany).

### 2.2. Cell Culture

Human liver cancer HepG2 cells were obtained from the Chinese Academy of Medical Sciences (Beijing, China). HepG2 cells were cultured in DMEM to be supplemented with 10% FBS, 100 *μ*g/mL streptomycin, and 100 U/mL penicillin. Cells were maintained at 37°C in a fully humidified atmosphere with 5% CO_2_-95% air.

### 2.3. Insulin Resistance Model in HepG2 Cells

Insulin resistance model was established as previous study described [17, 18], with minor modifications. HepG2 cells (2×10^4^ per well) were seeded in 96-well plates for 24 h. After cells adhere to the well, the medium was replaced with fresh medium with 5×10^−6^ mol/L insulin. Then cells were cultured for 36 h.

### 2.4. Sample Preparation

Pinobanksin, galangin, chrysin, and pinocembrin were dissolved in DMSO and diluted in DMEM containing 10% FBS, 100 *μ*g/mL streptomycin, and 100 U/mL penicillin. The final quantity of DMSO did not exceed 0.1% of culture media for all experiments.

### 2.5. MTT Assay

MTT assay was carried out as described by Mosmann [19], with minor modifications. HepG2 cells (2×10^4^ per well) were seeded in 96-well plates for 24 h. After discarding the medium, fresh medium was added containing various concentrations of pinobanksin (0, 4, 8, 16, 32, 64, and 128 *μ*mol/L), galangin (0, 4, 8, 16, 32, 64, and 128 *μ*mol/L), chrysin (0, 1, 2, 4, 8, 16, and 32 *μ*mol/L), or pinocembrin (0, 1, 2, 4, 8, 16, and 32 *μ*mol/L) and each concentration was repeated six times. After 24 h, the medium was discarded and cells were washed with PBS twice. Cells were then incubated with 100 *μ*L of 0.5 mg/mL MTT for 4 h in the dark. The supernatant was removed and 150 *μ*L of DMSO was added to completely solubilize formazan. The absorbance of each well was measured at 490 nm with a microplate reader. Cell viability was expressed as a percentage of the OD value of each treatment group relative to the OD value of control group.

### 2.6. Detection of Glucose Consumption

Glucose consumption was tested using a glucose assay kit, according to the manufacturer's instructions. In accordance with 2.3, insulin resistance model of HepG2 was established in 96-well plates. The cells were washed by PBS for two times, then the mediums were added containing various concentrations of pinobanksin (0, 4, 8, 16, and 32 *μ*mol/L), galangin (0, 10, 20, 40, and 80 *μ*mol/L), chrysin (0, 0.5, 1, 2, and 4 *μ*mol/L), or pinocembrin (0, 0.5, 1, 2, and 4 *μ*mol/L). Each concentration was repeated six times and the control group did not do any treatment. After 24 h, the glucose content of supernatant was measured by glucose assay kit. The absorbance of each well was measured at 505 nm with a microplate reader. Cell viability was determined by the MTT method of 2.5 to correct the differences of glucose measurement results due to the difference in cell numbers.

### 2.7. Detection of Glycogen Content

Intracellular glycogen was measured using a glycogen assay kit, according to the manufacturer's instructions. In accordance with 2.3, the HepG2 model was established in 6-well plates. Various concentrations of galangin (0, 10, 20, 40, and 80 *μ*mol/L) or pinocembrin (0, 0.5, 1, 2, and 4 *μ*mol/L) were added to each well and each concentration was repeated in six wells. After 24 h, the supernatant of each well was aspirated and the cell lysate was added to lyse the cells. The protein content of each well was determined by bicinchoninic acid protein assay kit. The content was expressed as mg/g protein.

### 2.8. Detection of Intracellular Hexokinase and Pyruvate Kinase

In accordance with 2.7, cells were treated and cultured. After incubation for 24 h, cells were collected in a centrifuge tube and the supernatant was discarded after centrifugation. The activities of hexokinase and pyruvate kinase were tested using hexokinase (HK) assay kit and pyruvate kinase (PK) assay kit, according to the manufacturer's instruction. The enzyme activity was calculated as follows:

(1) HK  activity  U/g  protein=1286×△Aprotein  contentPK  activity  U/g  protein=3216×△Aprotein  content

△A is absorbance change within five minutes.

### 2.9. Akt/mTOR Signaling Proteins Multiplex Analysis

In accordance with 2.7, cells were treated and cultured for 24 h. According to the MILLIPLEX® _MAP_ Akt/mTOR 11-plex Magnetic Bead Kit instructions, the lysed cells were collected as samples and the multiplex assay was performed in 96-well plates. The 96-well plates were washed with 100 *μ*L assay buffer, then 25 *μ*L of controls (or sample) and 25 *μ*L of beads were added in 96-well plates. Plates were incubated overnight at 4°C in the dark. In the next day, plates were washed with 100 *μ*L assay buffer twice. Then plates were incubated in biotinylated detection antibody cocktail at room temperature (RT) for 1 h. After removing the detection antibody cocktail, 25 *μ*L streptavidin-phycoerythrin (SAPE) was added in plates at RT for 15 minutes. Cell signaling amplification buffer was added and plates were incubated at RT for another 15 minutes. Finally, the SAPE and amplification buffer were replaced with 150 *μ*L assay buffer. All incubation steps were performed on a plate shaker at 100–300 rpm. Mean fluorescence intensity (MFI) data of assay plates were read and analyzed on a Luminex 200™ system. Data are normalized to phosphorylated protein/total protein.

### 2.10. Molecular Docking between Galangin/Pinocembrin and Human Insulin Receptor (IR)

Molecular docking was performed to investigate binding mode between galangin (or pinocembrin) and human insulin receptor (IR) using Autodock Vina 1.1.2 [20]. The three-dimensional (3D) structure of the human IR (PDB ID: 2HR7) was downloaded from RCSB Protein Data Bank (http://www.rcsb.org/pdb/home/home.do) [21]. The 2D structures of the galangin and pinocembrin were drawn by ChemBioDraw Ultra 14.0 and converted to 3D structures by the ChemBio3D Ultra 14.0 package [22]. The AutoDockTools 1.5.6 package [23, 24] was employed to generate the docking input files. The search grid of the IR was identified as center x: 16.322, center y: 35.12, and center z: 56.713 with dimensions size x: 15, size y: 15, and size z: 15. The value of exhaustiveness was set to 20. For Vina docking, the default parameters were used if it was not mentioned. The best-scoring pose as judged was chosen by the Vina docking score and visually analyzed using PyMoL 1.7.6 software (http://www.pymol.org/) [22].

### 2.11. Statistical Analysis

All the data were evaluated by GraphPad Prism 7. Values were indicated as the mean ± standard error mean (SEM). The statistical analysis included one-way ANOVA. A *p* value of <0.05 was considered statistically significant.

## 3. Result

### 3.1. Galangin and Pinocembrin Increased the Glucose Uptake of Insulin-Resistant HepG2 Cells

**Figure 1:**
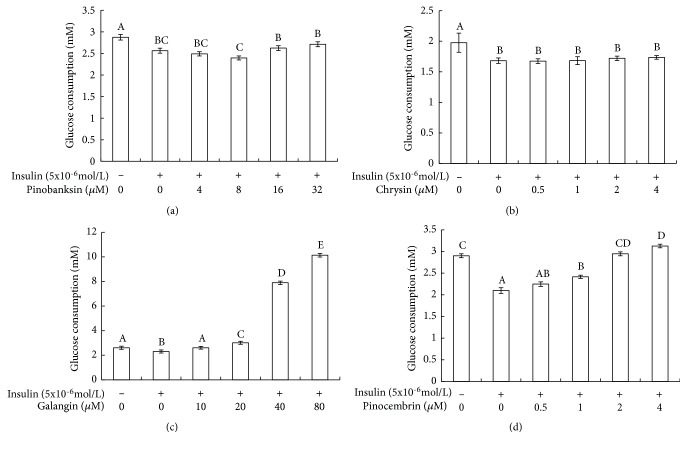
Effect of four flavones on glucose consumption in insulin-resistant HepG2 cells. Note: (1) (a) pinobanksin, (b) chrysin, (c) galangin, and (d) pinocembrin. (2) HepG2 cells were plated in 96-well plates overnight, followed by treated in the absence or presence of 5x10^−6^mol/L insulin. After 36 h, cells were exposed to different concentrations of flavonoids for 24h. Then the amount of glucose consumption in the HepG2 cells was detected by the glucose detection kit. (3) Values are means ± SEM from six separate determinations. Values with different letters (A–E) in the same column are significantly different from each other (p < 0.05).

In order to investigate whether four flavonoids have a positive influence on insulin resistance, we analyzed their effects on glucose uptake in insulin-resistant HepG2 cells. Glucose consumption is shown in Figure 1. As can be seen, insulin stimulation groups significantly decreased cellular glucose uptake in comparison with control groups (p < 0.05). Another notable result is that the glucose uptake had the wide variations in insulin stimulation groups after treatment using different concentrations of galangin and pinocembrin. The glucose uptake of the 80 *μ*M galangin-treated cells was about five times than that of insulin stimulation groups. Pinocembrin treatment was able to promote glucose uptake of insulin stimulation groups. The glucose uptake of the 4 *μ*M pinocembrin-treated cells was more 50% than that of insulin stimulation groups. However, there were no significant differences in the amount of glucose uptake of insulin stimulation groups after treated with pinobanksin and chrysin.

### 3.2. Galangin and Pinocembrin Promoted the Glycogen Synthesis of Insulin-Resistant HepG2 Cells

**Figure 2:**
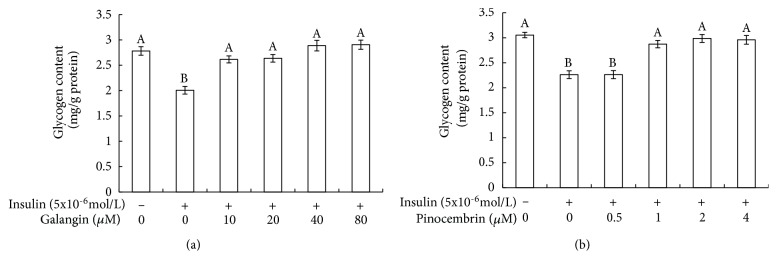
Effect of galangin and pinocembrin on glycogen content in insulin-resistant HepG2 cells. Note: (1) (a) galangin; (b) pinocembrin. (2) HepG2 cells were plated in 96-well plates overnight, followed by treated in the absence or presence of 5x10-6mol/L insulin. After 36 h, cells were exposed to different concentrations of galangin and pinocembrin for 24h. Then the amount of glycogen content in the HepG2 cells was detected by the glycogen detection kit. (3) Values are means ± SEM from six separate determinations. Values with different letters (A–B) in the same column are significantly different from each other (p < 0.05).

Glycogen synthesis is one of the main ways in liver to regulate glucose metabolism. Glycogen contents of HepG2 cells were presented in Figure 2. According to the Figure 2, compared with control groups, the glycogen content of insulin stimulation groups was significantly decreased (p<0.05). In contrast to insulin stimulation groups, galangin and pinocembrin appreciably promoted glycogen synthesis. Both 80 *μ*M galangin and 4 *μ*M pinocembrin treatment made the glycogen content of insulin stimulation groups to increase by as many as 50% and 30%, respectively.

### 3.3. Galangin and Pinocembrin Enhanced of Hexokinase and Pyruvate Kinase Activities

**Figure 3:**
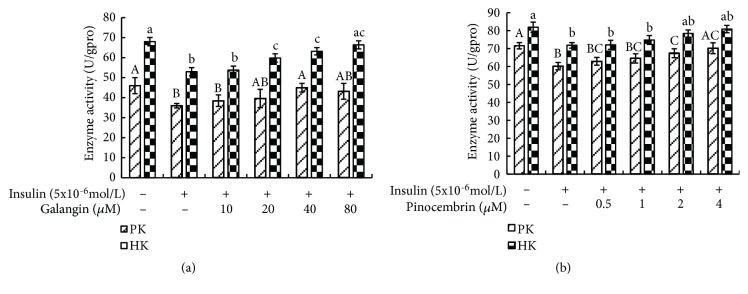
Effect of galangin and pinocembrin on hexokinase (HK) and pyruvate kinase (PK) activity in insulin-resistant HepG2 cells. Note: (1) (a) galangin; (b) pinocembrin. (2) HepG2 cells were plated in 96-well plates overnight, followed by treated in the absence or presence of 5x10-6mol/L insulin. After 36 h, cells were exposed to different concentrations of galangin and pinocembrin for 24h. Then the activity of intracellular hexokinase and pyruvate kinase was tested by hexokinase and pyruvate kinase assay kits. (3) Values are means ± SEM from six separate determinations. Values with different letters (A-C, a–c) in the same column are significantly different from each other (p < 0.05).

In order to investigate the effects of galangin and pinocembrin on glucose metabolism, the activities of hexokinase and pyruvate kinase were determined. As revealed in Figure 3, compared with control groups, the activities of hexokinase and pyruvate kinase were significantly repressed by insulin stimulation (p<0.05). However, this effect was substantially ameliorated by galangin and pinocembrin treatment. When 80 *μ*M galangin was present, the activities of hexokinase and pyruvate kinase were increased by 21.94% and 29.12%, respectively, compared with insulin stimulation groups.

### 3.4. Galangin Regulated Phosphorylation Levels of Key Akt/mTOR Signaling Proteins

**Figure 4:**
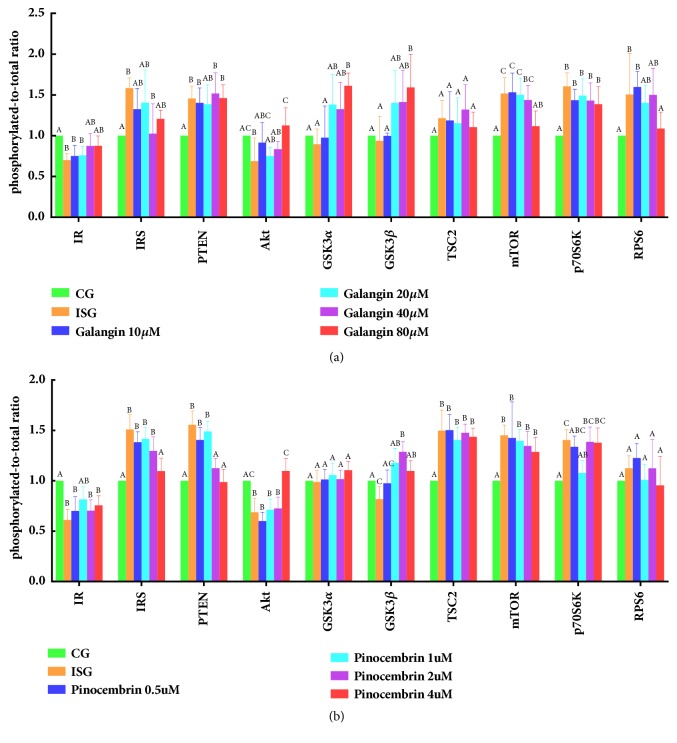
Effect of galangin and pinocembrin on the phosphorylation levels of key proteins of Akt/mTOR signaling pathway in insulin-resistant HepG2 cells. Note: (1) the CG is control groups; the ISG is insulin stimulation groups; (a) the 10 to 80 is concentration of galangin; (b) the 0.5 to 4 is concentration of pinocembrin. (2) The x-axis is the name of the key protein of Akt/mTOR signaling pathway. The y-axis is the phosphorylation level of the key protein of Akt/mTOR signaling pathway. (3) Values are means ± SEM from six separate determinations. Values with different letters (A–C) in the same column are significantly different from each other (p < 0.05).

To investigate whether galangin can get insulin resistance better, we examined the phosphorylation state of ten proteins on the Akt/mTOR signal pathway, including IR, IRS1, PTEN, Akt, GSK3*α*, GSK3*β*, TSC2, mTOR, p70S6K, and RPS6. Phosphorylation levels of ten protein are shown in Figure 4(a). According to the Figure 4(a), the phosphorylation levels of IR and Akt of insulin stimulation groups were remarkably lower than those of control groups (p<0.05). Compared with insulin stimulation groups, phosphorylation levels of IR, Akt, GSK3*α*, and GSK3*β* significantly increased (p<0.05) after galangin treatment. The phosphorylation level of Akt in the 80 *μ*M galangin-treated cells was 68% higher than that of insulin stimulation groups. On the other hand, insulin stimulation noticeably elevated the phosphorylation levels of IRS, PTEN, mTOR, p70s6K, and RPS6 in comparison to control groups (p<0.05), while galangin treatment significantly reduced IRS, mTOR, and RPS6 levels (p<0.05). Compared with insulin stimulation groups, the phosphorylation level of mTOR reduced by 37.84% after galangin treatment.

### 3.5. Pinocembrin Regulated Phosphorylation of Key Akt/mTOR Signaling Proteins

In order to further confirm pinocembrin's influence on the alleviation of insulin resistance, the phosphorylation of key Akt/mTOR signaling proteins was assayed in insulin-resistant HepG2 cells after pinocembrin treatment. Wide variations of phosphorylation levels were revealed in Figure 4(b). As can be seen, insulin stimulation could promote the phosphorylation of IRS, PTEN, TSC2, mTOR, and p70S6K, whereas pinocembrin treatment remarkably decreased phosphorylation of IRS, PTEN, and p70S6K. Moreover, after pinocembrin treatment, the phosphorylation levels of TSC2, mTOR, and RPS6 did almost not improve. The phosphorylation levels of IR, Akt, and GSK3*β* noticeably decreased in insulin stimulation groups. The phosphorylation of Akt seem to tend to increase dose-dependently with pinocembrin treatment.

### 3.6. Interaction between Galangin/Pinocembrin and Human Insulin Receptors

**Figure 5:**
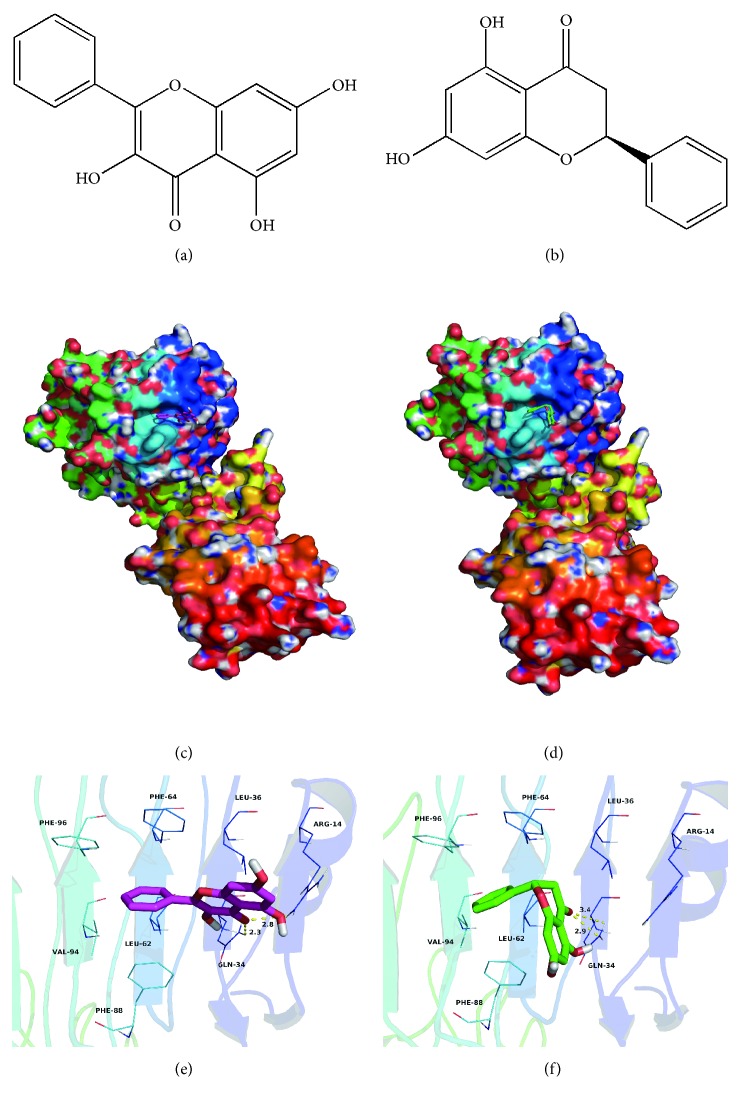
Molecular docking between galangin/pinocembrin and human insulin receptor. Note: (a) is the chemical structure of galangin. (b) is the chemical structure of pinocembrin. (c) shows the results of galangin docking with human insulin receptor. (d) shows the results of pinocembrin docking with human insulin receptor. (e) is a visual analysis of galangin docking with the human insulin receptor. (f) is a visual analysis of pinocembrin docking with the human insulin receptor.

Insulin receptor is embedded on the cell surface and specifically binds to insulin to activate the subsequent signaling pathway. We suspect that galangin and pinocembrin may firstly bind to the insulin receptor. Therefore, we used molecular docking to investigate their binding sites of the human IR. Theoretical binding modes are illustrated in Figure 5. Galangin and pinocembrin adopted a compact conformation in the binding pocket of the IR (Figures 5(c) and 5(d)). The phenyl groups of galangin and pinocembrin were surrounded by the residues Leu-62, Phe-64, Phe-88, Val-94, and Phe-96 at the hydrophobic pocket of the IR, forming a strong hydrophobic binding (Figures 5(e) and 5(f)). The phenyl groups of galangin and pinocembrin formed CH-*π* interactions with the side chains of the residues Phe-64, Phe-88, and Phe-96 (Figures 5(e) and 5(f)). Moreover, a cation-*π* interaction was observed between the side chain of the residue Arg-14 of IR and the 4H-chromen-4-one scaffold of the galangin, or the chroman-4-one scaffold of the pinocembrin, respectively. Importantly, the carbonyl “O” of the galangin formed two hydrogen bonds with the residues Arg-14 and Gln-34, with the lengths of 2.8 and 2.3 Å, respectively (Figure 5(e)). In comparison, the carbonyl “O” of the pinocembrin formed two hydrogen bonds with the residue Gln-34, with the lengths of 2.9 and 3.4 Å, respectively (Figure 5(f)).

## 4. Discussion

Insulin is a protein hormone secreted by islet beta cells, when the cells were stimulated by endogenous or exogenous substances such as glucose, lactose, ribose, arginine, and glucagon. Insulin is the only hormone in the body to lower blood glucose, and to promote glycogen, fat, and protein synthesis. Insulin resistance represents a decreased sensitivity and reactivity of target tissues to insulin in maintaining the balance and stability of body's glucose level. Therefore, insulin resistance can decrease glucose consumption and glycogen synthesis. In recent years, natural products have drawn more and more attention due to their positive effects in the treatment of insulin resistance, for example, cocoa [25], onion [26],* Tetrastigma obtectum* [27], and propolis [28]. Prior work has documented the effectiveness of propolis in reducing blood glucose levels and ameliorating insulin resistance in organism; Wataru Aoi, for example, reports that dietary propolis reduced blood glucose levels in Otsuka Long-Evans Tokushima Fatty rats and improved insulin sensitivity in the early stage of insulin resistance development [28]. However, these studies have not focused on the impact of individual propolis compounds on the signaling pathways related to insulin resistance. In this study, we tested the effect of galangin and pinocembrin in propolis on glucose uptake and Akt/mTOR signaling in HepG2 cells.

We find that, in virtually all cases, galangin and pinocembrin seem to have a significant role in promoting glucose metabolism. Our results reveal that galangin and pinocembrin can significantly increase glucose consumption (Figure 1) and glycogen synthesis (Figure 2) by enhancing the activities of hexokinase and pyruvate kinase (Figure 3). Moreover, the consumed glucose may have partially synthesized glycogen. Previous research has noted that hexokinase and pyruvate kinases play a crucial role in glucose metabolism [29, 30]. The current study is consistent with Hu et al., who discovered that the activities of hexokinase and pyruvate kinase were reduced in insulin resistance cells [31]. Our findings also confirmed that galangin dose-dependently normalized blood glucose in a fructose-induced rat model [32]. Most notably, we reveal a novel finding that pinocembrin with the lower concentrations can promote glucose metabolism and glycogen synthesis in insulin resistance cells.

**Figure 6:**
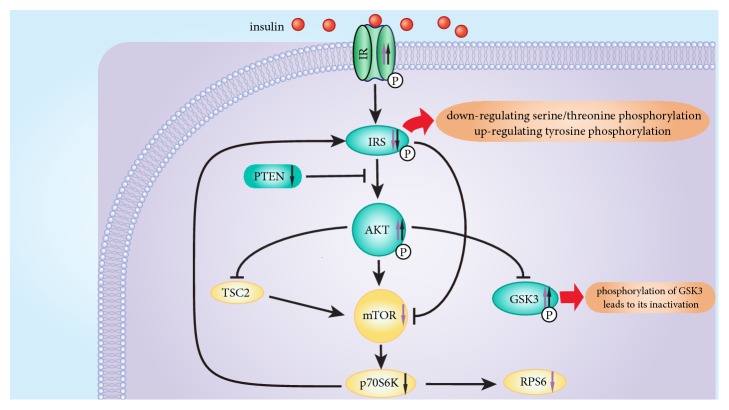
Overview of the Akt/mTOR pathway and regulatory effects of galangin and pinocembrin on the phosphorylation of key proteins.* Note*. “Purple color ↑”: galangin upregulates phosphorylation. “Black color ↑”: pinocembrin upregulates phosphorylation. “Purple color ↓”: galangin downregulates phosphorylation. “Black color ↓”: pinocembrin downregulates phosphorylation.

We hypothesize that galangin and pinocembrin may have a synergistic effect on the alleviation of insulin resistance via Akt/mTOR signaling pathway. Galangin and/or pinocembrin impact the phosphorylation of IR, IRS, PTEN, Akt, GSK3*α*, GSK3*β*, mTOR, p70S6K, and RPS6 (Figure 6). Both of galangin and pinocembrin can upregulate the phosphorylation of IR, Akt and GSK3*β* and downregulate the phosphorylation of IRS (Figure 6). Insulin can bind to the alpha subunit of IR and alters the configuration of the beta subunit, activating tyrosine protein kinases. Galangin and pinocembrin upregulated the phosphorylation levels of IR, to enhance its sensitivity and reactivity (Figure 6). In other words, galangin and pinocembrin can increase tyrosine protein kinase activity. In addition, serine/threonine phosphorylation of IRS reduces its tyrosine phosphorylation, and decreased tyrosine phosphorylation results in severe impairment of insulin signal transduction [33]. Galangin and pinocembrin treatment downregulated the serine/threonine phosphorylation of IRS (Figure 4). Tyrosine protein kinases can phosphorylate the tyrosine residues of IRS and in turn activate IRS. Activated IRS can promote the transmission of downstream signals [34]. Akt is an inducible downstream effector of IRS and an adaptor protein in Akt/mTOR signaling pathway [35]. In this study, we find that galangin and pinocembrin treatment upregulated the phosphorylation of Akt (Figure 6). The serine/threonine of Akt can be phosphorylated by tyrosine phosphorylated IRS, and serine phosphorylation site is the primary mechanism of insulin stimulates Akt activation [36]. Activated Akt transfers to cytoplasm or nucleus to continue the phosphorylation of subsequent substrates, thereby regulating glucose metabolism. On the one hand, activated Akt can activate glycogen synthase by upregulating phosphorylation of GSK3, thereby increasing glycogen synthesis and glucose consumption [37]. We find that galangin and pinocembrin can also upregulate the phosphorylation level of GSK3*β* in insulin-resistant HepG2 cells (Figure 6). Galangin can upregulate the phosphorylation of GSK3*α* (Figure 4(a)). Our findings confirm that galangin promotes phosphorylation of GSK3 to lead to its inactivation to induce type 2 diabetes [37]. On the other hand, activated Akt can directly phosphorylate the ser2448 site of mTOR. We find that high insulin treatment triggered insulin resistant by the overexpression of IRS and mTOR, and galangin treatment downregulated IRS and mTOR phosphorylation (Figure 4(a)). This result is consistent with the conclusions that the activity of mTOR can be inhibited by downregulating the serine phosphorylation of IRS and upregulating its tyrosine phosphorylation [38]. Activated mTOR phosphorylates p70S6K, one of the most studied substrates of mTOR. Although phosphorylation of p70S6K is able to contribute to the conduction of insulin signaling, overexpression of p70S6K triggers insulin resistance [39]. Our results indicate that pinocembrin downregulated the phosphorylation of p70S6K (Figure 4(b)). Dephosphorylation of p70S6K may cause a decrease in IRS serine phosphorylation and an increase in its tyrosine phosphorylation, resulting in upregulating Akt phosphorylation to alleviate insulin resistance [39]. Some studies have shown that RPS6 was involved in maintaining glucose stability and insulin sensitivity [40]. Our results indicate that galangin treatment downregulated phosphorylation of RPS6 (Figure 4(a)) to inhibit overexpression of RPS6 phosphorylation. We speculate that overexpression of RPS6 phosphorylation may inhibit the synthesis of glycometabolism enzymes, thereby reducing the utilization of glucose. Overall, we hypothesize that galangin and pinocembrin may synergistically relieve insulin resistance through regulating the protein phosphorylation of key Akt/mTOR signal proteins.

Insulin receptor is a tetramer between two alpha subunits and two beta subunits, together linked by disulfide bonds. Two alpha subunits are located on the outer side of the plasma membrane with insulin binding sites; the two beta subunits are transmembrane proteins acting as signal transducers. The specific binding of insulin to insulin receptor can promote subsequent signal transduction [41]. In this study, we provide theoretical binding modes between galangin or pinocembrin and human insulin receptor. Our results demonstrate that galangin and pinocembrin can alter insulin receptor conformation by binding to the insulin receptor, thereby increasing insulin receptor sensitivity and insulin availability (Figure 5). Moreover, there is a difference in docking structure between galangin/pinocembrin and insulin receptors. This result is also further confirmed that galangin and pinocembrin can regulate different Akt/mTOR signaling proteins by binding to the insulin receptor, thus relieving insulin resistance.

## 5. Conclusions

Pinobanksin and chrysin are ineffective for promoting glucose metabolism. On the contrary, galangin and pinocembrin ameliorate insulin resistance by increasing the activity of hexokinase and pyruvate kinase, promoting glucose consumption and glycogen synthesis. We also report here for the first time that pinocembrin has the effect of alleviating insulin resistance. In addition, we hypothesize that galangin and pinocembrin may have a synergistic effect via Akt/mTOR signaling pathway, while galangin and pinocembrin upregulate the phosphorylation of IR, Akt, and GSK3*β*, downregulate the phosphorylation of IRS, and activate Akt/mTOR pathway. These results appear to provide a reliable evidence to develop novel propolis nutraceuticals directed at insulin resistance.

## Data Availability

The data used to support the findings of this study are included within the article.

## Abbreviations

DMEM: Dulbecco's modified eagle medium
PBS: Phosphate buffered saline
HK: Hexokinase
PK: Pyruvate kinase
DMSO: Dimethyl sulfoxide
MTT: 3-(4,5-Dimethyl-2-thiazolyl)-2,5-diphenyl-2-H-tetrazolium bromide
FBS: Fetal bovine serum
IR: Insulin receptor
IRS: Insulin receptor substrate
Akt: Protein kinase B
GSK3: Glycogen synthetic kinase 3
mTOR: Mechanistic target of rapamycin
p70S6K: Ribosomal protein s6 kinase, 70kda
RPS6: Ribosomal protein s6
3D: Three-dimensional.
